# Evaluation of sex differences in survival among glioblastoma patients treated with immune checkpoint inhibitors

**DOI:** 10.1093/noajnl/vdaf250

**Published:** 2025-12-08

**Authors:** Vihang Nakhate, Catharina Westergaard, Zhou Lan, Aleksandra B Lasica, Alona Muzikansky, Brooke Barlow, Alyssa Russ, Loai Aker, Albert Jiao, Ian Pan, Thomas A Nelson, Chibueze D Nwagwu, Elisa Aquilanti, Tracy T Batchelor, Rameen Beroukhim, Tamar R Berger, Ugonma Chukwueke, L Nicolas Gonzalez Castro, Eudocia Quant Lee, J Ricardo Mcfaline-Figueroa, Lakshmi Nayak, John Y Rhee, David A Reardon, Raymond Y Huang, Patrick Y Wen, Gilbert Youssef

**Affiliations:** Center for Neuro-Oncology, Dana-Farber Cancer Institute, Boston; Harvard Medical School, Boston; Center for Neuro-Oncology, Dana-Farber Cancer Institute, Boston; Harvard Medical School, Boston; Center for Clinical Investigation, Brigham and Women’s Hospital, Boston; Center for Neuro-Oncology, Dana-Farber Cancer Institute, Boston; Harvard Medical School, Boston; Biostatistics Center, Massachusetts General Hospital, Boston; Center for Neuro-Oncology, Dana-Farber Cancer Institute, Boston; Center for Neuro-Oncology, Dana-Farber Cancer Institute, Boston; Harvard Medical School, Boston; Division of Neuroradiology, Department of Radiology, Brigham and Women’s Hospital, Boston; Harvard Medical School, Boston; Division of Neuroradiology, Department of Radiology, Brigham and Women’s Hospital, Boston; Harvard Medical School, Boston; Division of Neuroradiology, Department of Radiology, Brigham and Women’s Hospital, Boston; Division of Neuro-Oncology, Department of Neurosurgery, University of California San Francisco, San Francisco; Harvard Medical School, Boston; Department of Neurosurgery, Brigham and Women’s Hospital, Boston; Center for Neuro-Oncology, Dana-Farber Cancer Institute, Boston; Harvard Medical School, Boston; Center for Neuro-Oncology, Dana-Farber Cancer Institute, Boston; Harvard Medical School, Boston; Department of Neurology, Brigham and Women’s Hospital, Boston; Center for Neuro-Oncology, Dana-Farber Cancer Institute, Boston; Harvard Medical School, Boston; Center for Neuro-Oncology, Dana-Farber Cancer Institute, Boston; Harvard Medical School, Boston; Center for Neuro-Oncology, Dana-Farber Cancer Institute, Boston; Harvard Medical School, Boston; Center for Neuro-Oncology, Dana-Farber Cancer Institute, Boston; Harvard Medical School, Boston; Department of Neurology, Brigham and Women’s Hospital, Boston; Center for Neuro-Oncology, Dana-Farber Cancer Institute, Boston; Harvard Medical School, Boston; Brain & Spine Tumor Center, Perlmutter Cancer Center, NYU Langone Health, New York; Department of Neurology and Medicine, Grossman School of Medicine, New York University, New York; Center for Neuro-Oncology, Dana-Farber Cancer Institute, Boston; Harvard Medical School, Boston; Center for Neuro-Oncology, Dana-Farber Cancer Institute, Boston; Harvard Medical School, Boston; Department of Supportive Oncology, Dana-Farber Cancer Institute, Boston; Center for Neuro-Oncology, Dana-Farber Cancer Institute, Boston; Harvard Medical School, Boston; Harvard Medical School, Boston; Division of Neuroradiology, Department of Radiology, Brigham and Women’s Hospital, Boston; Center for Neuro-Oncology, Dana-Farber Cancer Institute, Boston; Harvard Medical School, Boston; Center for Neuro-Oncology, Dana-Farber Cancer Institute, Boston; Harvard Medical School, Boston

**Keywords:** glioblastoma, immune checkpoint inhibitors, immunotherapy, sex differences

## Abstract

**Background:**

Sex differences in glioblastoma (GBM) are recognized, but their treatment implications remain unclear. Recent preclinical studies have characterized mechanisms of sex-biased anti-tumor immunity in GBM, and have found in murine models that males derive greater survival benefit from immune checkpoint inhibitor (ICI). We evaluated sex differences associated with ICI in GBM patients.

**Methods:**

We retrospectively evaluated consecutive patients with newly diagnosed GBM (nGBM) or recurrent GBM (rGBM) treated with ICI on clinical trials at one institution from 2014 to 2022. Progression-free survival (PFS) and overall survival (OS) were evaluated by Kaplan-Meier analysis, univariate and multivariable regression models. Sex-by-treatment interactions were assessed relative to a concurrent reference group treated on non-ICI clinical trials.

**Results:**

296 patients with nGBM (58% male, 19% ICI) and 458 patients with rGBM (60% male, 40% ICI) were evaluated. In nGBM, ICI was not associated with sex difference in PFS (HR_male_ 1.35; 95% CI, 0.62–2.95; *P* = .446; *P*_interaction_ = .142) or OS (HR_male_ 1.15 [0.53–2.53], *P* = .722; *P*_interaction_ = .438) compared to non-ICI treatment. In rGBM, males receiving ICI had worse OS (HR_male_ 1.64 [1.09–2.47], *P* = .017) and trended towards worse PFS (HR_male_ 1.41 [0.94–2.11], *P* = .095), but sex differences with ICI were not significantly different compared to non-ICI treatment (PFS *P*_interaction_ = .610; OS *P*_interaction_ = .361). No sex differences were observed when all immunotherapies were analyzed collectively.

**Conclusion:**

In nGBM and rGBM, ICI therapy was not associated with sex difference in PFS or OS. Clinically meaningful sex-based outcome differences may be better understood by prospective evaluation in clinical trials.

Key Points

- ICI therapy in GBM patients was not associated with sex-based differences in PFS or OS
- Immunotherapy treatments collectively in GBM patients were not associated with sex-based differences in PFS or OS

Importance of the study

There is growing interest in understanding sex differences in glioblastoma (GBM) and their implications for treatment. Recent preclinical murine studies have characterized sex-based differences in GBM anti-tumor immunity. Notably, immune checkpoint inhibitor (ICI) therapy in GBM mouse models conferred survival benefit to male but not female mice. Whether ICI therapy confers sex-specific survival benefit in GBM patients has not been evaluated. In a large cohort of GBM patients, we found that ICI was not associated with sex differences in progression-free survival or overall survival. Our study highlights the multifactorial nature of sex differences in GBM patients, the importance of evaluating preclinical findings in clinical studies, and the need for prospective assessment of sex-based differences in clinical trials.

Sex-based differences in glioblastoma (GBM) have been recognized, and there is growing interest in understanding their underlying mechanisms and potential treatment implications. The incidence of GBM is 1.6 times higher in males relative to females.^1^ Additionally, population-based studies have found that females with GBM have increased survival compared to males.^2^^,^^3^ Mechanisms that may drive these differences remain incompletely understood though immunologic, hormonal, and metabolic contributors have been proposed.^4^

Recent preclinical studies have found sex differences in GBM anti-tumor immunity that may drive differential outcomes.^5^^,^^6^ Glioblastoma has an immunosuppressive tumor microenvironment characterized by infiltration of myeloid cell populations, with associated reduction in circulating T-cells and weakened systemic anti-tumor immunity.^7^^,^^8^ Differential sex-based localization of myeloid-derived suppressor cell (MDSC) subtypes has been reported in preclinical models—increased monocytic MDSCs in the male GBM microenvironment and increased granulocytic MDSCs in the female peripheral blood.^5^ In a recent preclinical study, sex-based T-cell exhaustion in GBM was associated with survival differences.^6^ Glioblastoma in male mice and in human male patients showed evidence of increased T-cell exhaustion relative to their female counterparts, mediated by the X-chromosome encoded UTX protein. Interestingly, treatment with anti-programmed cell death protein 1 (anti-PD1) antibody extended survival in male mice relative to controls but conferred no benefit to females, suggesting a differential sex-based response to immune checkpoint blockade.^6^ Whether immune checkpoint inhibitor (ICI) therapy confers sex-specific survival benefit in GBM patients has not previously been evaluated.

As ICI therapy is not approved by the Food and Drug Administration (FDA) for GBM, its use is largely limited to the clinical trial setting. We retrospectively evaluated whether ICI treatment on clinical trials for patients with newly diagnosed GBM (nGBM) or recurrent GBM (rGBM) was associated with sex differences in survival outcomes.

## Methods

### Patient Selection

We retrospectively identified consecutive patients with nGBM or rGBM who enrolled on clinical trials at Dana-Farber Cancer Institute (DFCI) between 2014 and 2022. Integrated diagnosis of GBM, *IDH* wild-type, was verified by review of histopathologic reports and molecular testing results, according to the 2021 World Health Organization Classification of Tumors of the Central Nervous System.^9^ Additional inclusion criteria were age of at least 18 years, supratentorial tumor location, and for nGBM patients receipt of standard-of-care radiation. Patients were divided into nGBM and rGBM cohorts. Patients enrolled in both nGBM and rGBM trials were included, as the two cohorts were analyzed separately. For patients who enrolled in multiple rGBM trials, only their first enrollment, corresponding to their earliest recurrence, was included to prevent duplicate representation in analyses.

Patients who received ICI treatment on trial were identified for analysis. The remaining patients who received non-ICI treatments were analyzed as a reference group. Among non-ICI treatments, immunotherapy treatments were identified for additional exploratory analyses. Treatments received are shown in Table S1 and S2. The study was approved by the DFCI Institutional Review Board. The requirement of informed consent was waived for this retrospective analysis of clinical data.

### Clinical Data Collection

Clinical trial records were reviewed to collect trial treatment information. Electronic medical records were reviewed to collect demographic, molecular, and clinical variables including potential confounders of outcomes associated with GBM or ICI therapy. Collected variables included biological sex, age at diagnosis, Karnofsky performance score (KPS) at diagnosis, O^6^-methylguanine methyltransferase (*MGMT)* promoter methylation status, extent of resection (EOR) in nGBM, pre-treatment tumor volume in rGBM, absolute lymphocyte count (ALC) at start of treatment and ALC nadir on treatment,^10^ dexamethasone dose at start of treatment and peak maintenance dexamethasone dose on treatment,^11^ receipt of standard-of-care concurrent and adjuvant temozolomide in nGBM, receipt of concurrent bevacizumab, and receipt of concurrent radiation in rGBM. Date of progression, as identified by the treating oncologist on trial, and date of death or date of last follow-up were collected. EOR in nGBM was determined by review of postoperative MRI for residual enhancing disease after surgery. Gross-total resection (GTR) was considered the absence of measurable enhancing residual disease, as defined in the RANO 2.0 criteria.^12^ Tumor volume in rGBM was determined as below.

### Tumor Volumetrics

Pre-treatment enhancing tumor volume was evaluated in the rGBM cohort, since not all patients underwent surgical resection at time of progression. Tumor volume was determined by volumetric measurements computed from the baseline pre-treatment MRI. For patients who received surgery at relapse or enrolled on a surgical window-of-­opportunity trial, postoperative pre-treatment MRIs were used. A 3D convolutional neural network was trained on multi-parametric MRI examinations (T1-weighted, T2-weighted, FLAIR, and post-contrast T1-weighted) from patients with treated high grade glioma to predict segmentation masks for residual enhancing tumor.^13^ A board-certified neuroradiologist then reviewed the automatically generated segmentation masks to make corrections as needed. Volumetric data was derived from finalized segmentation masks using the MRI voxel size as determined by imaging metadata. In the multivariable analyses, tumor volume was classified into ≤1 cm^3^ or >1 cm^3^ as the prognostic threshold for survival.^14^

### Statistical Analyses

Summary statistics were calculated for pertinent demographic, molecular and clinical variables. Differences between groups were assessed using Pearson’s chi-square test for categorical variables or Wilcoxon rank-sum test for continuous variables. Primary survival endpoints were progression-free survival (PFS) and overall survival (OS). Progression-free survival was defined as time in months between treatment start date and date of progression. Overall survival was defined as time in months between treatment start date and date of death. Patients were right-censored for loss to follow-up or for being event-free by the data cutoff date of April 1, 2025. Treatment start date was considered the first date of radiation for nGBM or the first date of trial treatment for rGBM. For rGBM surgical window-of-opportunity trials, the first date of postoperative treatment was used. Progression-free survival and Overall survival were assessed by the Kaplan-Meier method. Sex differences in outcomes were assessed with univariate Cox proportional hazards models for biological sex and using log-rank tests. Multivariable Cox proportional hazards models were used to evaluate sex differences while adjusting for relevant potential confounders. Hazard ratios (HR) are reported for males, with females as the reference group. Cox proportional hazard models with an interaction term between ICI and sex were used to evaluate sex differences between ICI and non-ICI treatment groups. This interaction analysis tested whether sex differences in outcomes were specific to ICI treatment rather than reflective of baseline differences observed in patients on non-ICI treatments. Survival data in the rGBM cohort violated the proportional hazards assumption, therefore weighted Cox regression was used to derive valid HRs and *P*-values.^15^^,^^16^ The threshold for statistical significance was set at *P*-value < .05, and 95% confidence intervals (CI) are reported. Statistical analyses were conducted using R 4.2.0.

## Results

### Patient Characteristics

The nGBM cohort (hereafter *nGBM*) comprised 296 patients consecutively enrolled in clinical trials. Patient characteristics are shown in Table 1. *nGBM* included 172 (58%) males and 124 (42%) females. Fifty-six patients (19%) received ICI, including 33 (59%) males and 23 (41%) females. ICI treatment included agents targeting PD1 (80%), programmed death-ligand 1 (PDL1) (16%), and combination PD1/cytotoxic T-lymphocyte-associated protein 4 (CTLA4) (4%) (Table S1). Median age was 60 years, and median KPS was 90. *MGMT* promoter was unmethylated in 220 (74%) and methylated in 64 (22%). One hundred ninety-one patients (65%) had GTR. ALC nadir was significantly lower in females than in males (Table 1).

**Table 1.** Patient characteristics in the newly diagnosed and recurrent glioblastoma cohorts.

	Newly diagnosed GBM				Recurrent GBM			
	Total	Male	Female	*P*-value	Total	Male	Female	*P*-value
**Patients**	296	172 (58%)	124 (42%)		458	274 (60%)	184 (40%)	
**Age** (years, IQR)	60 (54, 66)	60 (53, 67)	60 (54, 66)	0.717	58 (51, 64)	59 (52, 64)	56 (50, 63)	0.025^*^
**KPS** (range)	90 (70,100)	90 (70, 100)	90 (70, 100)	0.893	80 (60, 100)	80 (60, 100)	80 (60, 100)	0.204
**MGMTp**								
*Unmethylated*	220 (74%)	127 (74%)	93 (75%)	0.622	270 (59%)	160 (58%)	110 (60%)	0.849
*Methylated*	64 (22%)	38 (22%)	26 (21%)		138 (30%)	82 (30%)	56 (30%)	
*Partially methylated*	10 (3%)	5 (3%)	5 (4%)		22 (5%)	13 (5%)	9 (5%)	
*Unknown*	2 (1%)	2 (1%)	0		28 (6%)	19 (7%)	9 (5%)	
**EOR**								
*Biopsy*	12 (4%)	4 (2%)	8 (6%)	0.200				
*STR*	93 (31%)	56 (33%)	37 (30%)					
*GTR*	191 (65%)	112 (65%)	79 (64%)					
**Tumor Volume**								
*≤ 1* *cm^3^*					42 (9%)	26 (10%)	16 (9%)	0.959
*> 1* *cm^3^*					411 (90%)	245 (89%)	166 (90%)	
*Unavailable*					5 (1%)	3 (1%)	2 (1%)	
**ICI treatment**								
*Yes*	56 (19%)	33 (19%)	23 (19%)	1.00	184 (40%)	114 (42%)	70 (38%)	0.506
*No*	240 (81%)	139 (81%)	101 (81%)		274 (60%)	160 (58%)	114 (62%)	
**IM treatment**								
*Yes*	105 (35%)	57 (33%)	48 (39%)	0.387	230 (50%)	139 (51%)	91 (49%)	0.864
*No*	191 (65%)	115 (67%)	76 (61%)		228 (50%)	135 (49%)	93 (51%)	
**Number of Relapse**								
* 1*					382 (83%)	232 (85%)	150 (82%)	0.447
* ≥2*					76 (17%)	42 (15%)	34 (18%)	
**Adjuvant TMZ**	153 (52%)	93 (54%)	60 (48%)	0.397				
**Bevacizumab**	4 (1%)	2 (1%)	2 (2%)	1.00	124 (27%)	76 (28%)	48 (26%)	0.778
**Radiation**	296 (100%)	172 (100%)	124 (100%)	1.00	42 (9%)	22 (8%)	20 (11%)	0.386
**Dex starting dose** (mg/day)	0 (0, 0)	0 (0, 0)	0 (0, 0)	0.482	0 (0, 2)	0 (0, 2)	0 (0, 2)	0.592
**Dex peak dose** (mg/day)	0 (0, 4)	0 (0, 4)	0 (0, 4)	0.859	2.0 (0, 4)	2 (0, 4)	2.0 (0, 4)	0.788
**ALC start** (×10^3^/µL)	1.3 (1.0, 1.8)	1.3 (1.0, 1.7)	1.4 (0.9, 1.8)	0.912	1.0 (0.7, 1.3)	1.0 (0.8, 1.3)	0.9 (0.6, 1.1)	<0.001*
**ALC nadir** (×10^3^/µL)	0.55 (0.34, 0.78)	0.64 (0.40, 0.84)	0.45 (0.31, 0.70)	<0.001*^∗^	0.67 (0.45, 0.89)	0.71 (0.50, 0.95)	0.58 (0.38, 0.81)	<0.001*

Abbreviations: ALC, absolute lymphocyte count; Dex, dexamethasone; EOR, extent of resection; GBM, glioblastoma; GTR, gross total resection; ICI, immune checkpoint inhibitor; IM, immunotherapy; IQR, interquartile range; KPS, Karnofsky performance status; MGMTp, O^6^-methylguanine methyltransferase promoter; STR, sub-total resection; TMZ, temozolomide.

Values are reported as number (%) or median (IQR), except KPS which is reported as median (range).

∗ denotes *P*-values < .05

The rGBM cohort (hereafter *rGBM*) comprised 458 patients, including 274 (60%) males and 184 (40%) females (Table 1). One hundred eighty-four patients (40%) received ICI on trial, including 114 (62%) males and 70 (38%) females. ICI treatment included agents targeting PD1 (81.5%), PDL1 (8%), lymphocyte activation gene 3 (LAG3) (1%), combination PD1/CTLA4 (9%), and combination LAG3/PD1 (0.5%) (Table S2). Median age in *rGBM* was 58 years, and median KPS was 80. Pre-treatment enhancing tumor volume was ≤1 cm^3^ in 42 (9%) patients. Age at diagnosis, ALC at the start of treatment, and ALC nadir were significantly lower in females than in males (Table 1).

### Newly Diagnosed GBM

**Figure 1.**
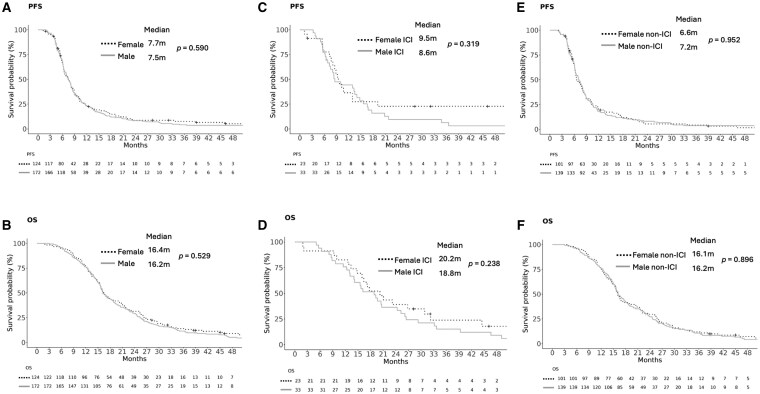
Kaplan-Meier analysis of progression-free survival (PFS) and overall survival (OS) in newly diagnosed glioblastoma, *IDH* wild-type. (A) PFS in all patients. (B) OS in all patients. (C) PFS in patients who received immune checkpoint inhibitor (ICI). (D) OS in patients who received ICI. (E) PFS in patients who received non-ICI treatment. (F) OS in patients who received non-ICI treatment. *P*-values from log rank tests are shown. Censored observations are denoted by “+” symbols. Abbreviations: IDH, isocitrate dehydrogenase; m, months.

In *nGBM* among all patients, there was no statistically significant sex-based difference in PFS or OS. Median PFS was 7.5 months for males and 7.7 months for females (HR for males 1.07; 95% CI, 0.84–1.36; *P* = .590) (Figure 1A). Median OS was 16.2 months for males and 16.4 months for females (HR 1.08; 95% CI, 0.85–1.37; *P* = .529) (Figure 1B). Among *nGBM* patients who received ICI, there was no statistically significant sex-based difference in PFS (HR 1.35; 95% CI, 0.75–2.45, *P* = .319) or OS (HR 1.42; 95% CI, 0.79–2.53; *P* = .238) (Figure 1C and D). Among patients who received non-ICI treatments, there was similarly no statistically significant sex-based difference in PFS (HR 1.01; 95% CI, 0.77–1.31; *P* = .952) or OS (HR = 1.02; 95% CI, 0.78–1.32; *P* = .896) (Figure 1E and F). There was no statistically significant interaction between sex and treatment for PFS (*P* = .370) or for OS (*P* = .318), consistent with no sex difference in survival outcomes with ICI treatment compared to non-ICI treatment.

**Figure 2.**
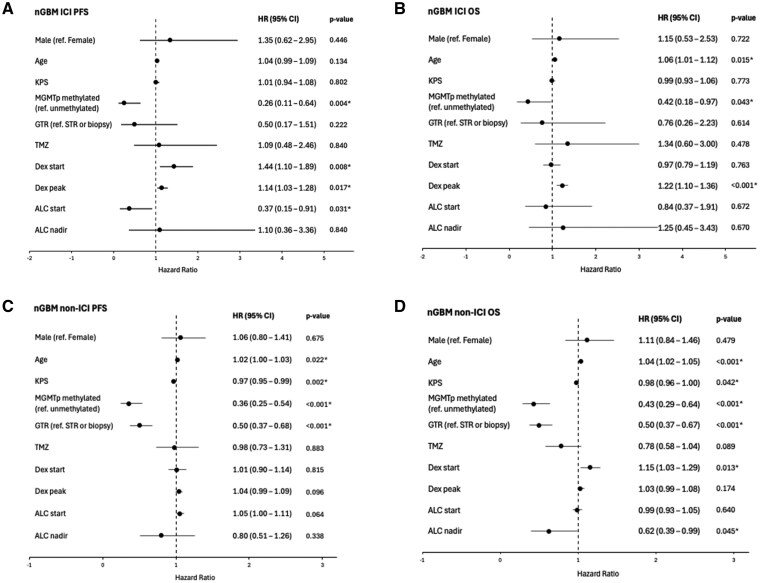
Forest plots of multivariable analyses for progression-free survival (PFS) and overall survival (OS) in newly diagnosed glioblastoma (nGBM), *IDH* wild-type. (A) PFS in patients who received immune checkpoint inhibitor (ICI). (B) OS in patients who received ICI. (C) PFS in patients who received non-ICI treatment. (D) OS in patients who received non-ICI treatment. P-values < 0.05 are denoted by asterisks (*). Abbreviations: ALC, absolute lymphocyte count; dex peak, dexamethasone peak dose (mg/day); dex start, dexamethasone dose (mg/day) at start of treatment; GTR, gross total resection; KPS, Karnofsky performance score; MGMTp, O6-methylguanine-DNA methyltransferase promoter; ref., reference; STR, sub-total resection; TMZ, temozolomide concurrent and adjuvant treatment.

Multivariable analyses adjusted for age, KPS, *MGMT* promoter methylation, EOR, receipt of concurrent and adjuvant temozolomide, dexamethasone starting and peak doses, starting ALC, and ALC nadir. Adjusting for these covariates, there was no statistically significant sex-based difference in PFS or OS in *nGBM* overall (Figure S1A and B). Among *nGBM* patients who received ICI, multivariable analysis showed no significant sex-based difference in PFS (HR 1.35; 95% CI, 0.62–2.95; *P* = .446) or OS (HR 1.15; 95% CI, 0.53–2.53; *P* = .722) (Figure 2A and B). Methylated *MGMT* promoter, lower starting and peak dexamethasone dose, and higher starting ALC were independently associated with decreased risk of progression (Figure 2A). Younger age, methylated *MGMT* promoter, and lower peak dexamethasone dose were independently associated with decreased risk of death (Figure 2B). No significant sex differences were found in multivariable analyses for *nGBM* patients who received non-ICI treatments (Figure 2C and D). No statistically significant interaction was found between treatment group and sex for PFS (*P* = .142) or OS (*P* = .438) in *nGBM* on multivariable analysis.

### Recurrent GBM

**Figure 3.**
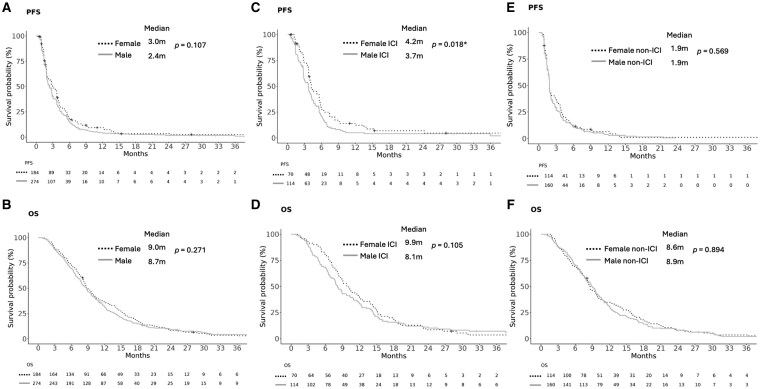
Kaplan-Meier analysis of progression-free survival (PFS) and overall survival (OS) in recurrent glioblastoma, *IDH* wild-type. (A) PFS in all patients. (B) OS in all patients. (C) PFS in patients who received immune checkpoint inhibitor (ICI). (D) OS in patients who received ICI. (E) PFS in patients who received non-ICI treatment. (F) OS in patients who received non-ICI treatment. P-values from log rank tests are shown. Censored observations are denoted by “+” symbols. P-values < 0.05 are denoted by asterisks (*). Abbreviations: IDH, isocitrate dehydrogenase; m, months.

In *rGBM* among all patients, there was no statistically significant sex-based difference in PFS or OS. Median PFS was 2.4 months for males and 3.0 months for females (HR for males 1.19; 95% CI, 0.96–1.48; *P* = .107) (Figure 3A). Median OS was 8.7 months for males and 9.0 months for females (HR 1.13; 95% CI, 0.91–1.40; *P* = .271) (Figure 3B). Among *rGBM* patients who received ICI, males had significantly worse PFS than females (HR 1.51; 95% CI, 1.1–2.1; *P* = .018) and a trend towards worse OS (HR 1.32; 95% CI, 0.94–1.84; *P* = .105) (Figure 3C and D). Among patients receiving non-ICI treatments on trial, there was no statistically significant sex-based difference in PFS (HR 1.08; 95% CI, 0.82–1.42; *P* = .569) or OS (HR 1.02; 95% CI, 0.77–1.35; *P* = .894) (Figure 3E andF). There was no statistically significant interaction between sex and treatment group for PFS (*P* = .427) or for OS (*P* = .993), consistent with no sex difference in survival outcomes with ICI treatment compared to non-ICI treatment.

**Figure 4.**
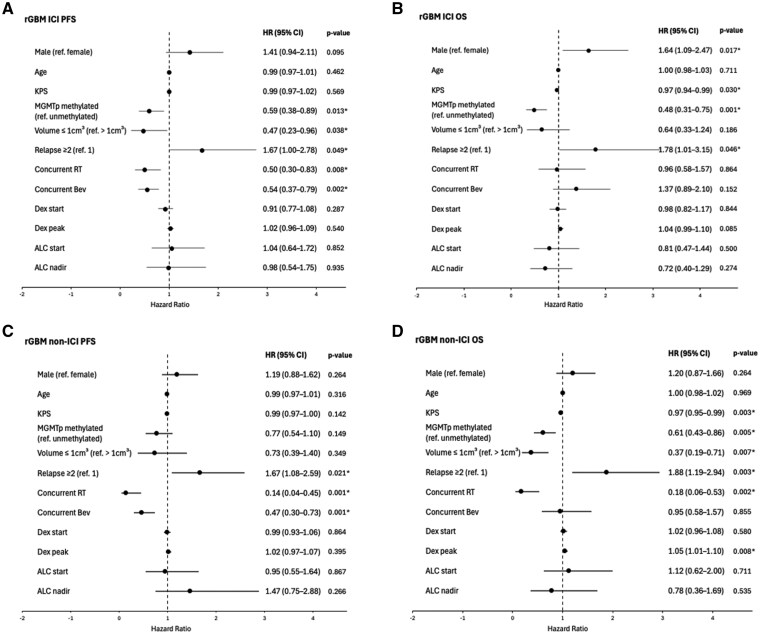
Forest plots of multivariable analyses for progression-free survival (PFS) and overall survival (OS) in recurrent glioblastoma (rGBM), *IDH* wild-type. (A) PFS in patients who received immune checkpoint inhibitor (ICI). (B) OS in patients who received ICI. (C) PFS in patients who received non-ICI treatment. (D) OS in patients who received non-ICI treatment. *P*-values < 0.05 are denoted by asterisks (*). Abbreviations: ALC, absolute lymphocyte count; Bev, bevacizumab; dex peak, dexamethasone peak dose (mg/day); dex start, dexamethasone dose (mg/day) at start of treatment; KPS, Karnofsky performance score; rGBM, recurrent glioblastoma; MGMTp, O6-methylguanine-DNA methyltransferase promoter; ref., reference; RT, radiation therapy.

On multivariable analysis adjusting for age, KPS, *MGMT* promoter methylation, pre-treatment tumor volume, concurrent radiation, concurrent bevacizumab, number of relapses, dexamethasone starting and peak doses, starting ALC and ALC nadir, there was a statistically nonsignificant trend towards worse PFS and OS in males compared to females in *rGBM* (Figure S1C and D). Among *rGBM* patients who received ICI, multivariable analysis showed significantly worse OS in males (adjusted HR 1.64; 95% CI, 1.09–2.47; *P* = .017) and a trend towards worse PFS in males (adjusted HR 1.41; 95% CI, 0.94–2.11; *P* = .095) (Figure 4A andB). Methylated *MGMT* promoter, pre-treatment tumor volume ≤1 cm³, first relapse, concurrent radiation, and concurrent bevacizumab were independently associated with decreased risk of progression (Figure 4A). Female sex, higher KPS, methylated *MGMT* promoter, and first relapse were independently associated with decreased risk of death (Figure 4B). No statistically significant sex differences were found in multivariable analyses for *rGBM* patients receiving non-ICI treatments (Figure 4C andD). No significant interaction was found between treatment group and sex for PFS (*P* = .610) or OS (*P* = .361) in *rGBM*.

#### Immunotherapy analyses

We conducted secondary analyses to evaluate whether these results extended beyond ICI treatment to immunotherapy collectively. Non-ICI immunotherapies in *nGBM* included peptide vaccine (14%) and anti-phosphatidylserine antibody (6%) (Table S1), and in *rGBM* included viral/gene/peptide-based immunotherapy (15%) and immunomodulators (2%, including IDO1 inhibitor, CSF1R inhibitor, or anti-CD137 antibody) (Table S2). There was no significant difference between the proportion of males and females who received immunotherapies (Table 1). We conducted similar univariate, multivariable, and interaction analyses to evaluate sex differences in PFS and OS using immunotherapy (IM) and non-immunotherapy (non-IM) treatment groups.

In both *nGBM* and *rGBM*, there were no statistically significant sex differences in PFS or OS associated with IM (Figure S2-S5). Among *nGBM,* univariate and multivariable analyses showed no statistically significant sex difference in either PFS or OS for the IM and non-IM treatment groups (Figure S2 and  S3). There was no significant interaction between sex and treatment group for PFS (*P* = .128) or OS (*P* = .385). Among *rGBM* patients who received IM, on univariate analyses males had significantly lower PFS (HR 1.42; 95% CI, 1.04–1.93; *P* = .026) and no significant difference in OS (HR 1.17; 95% CI, 0.87–1.58; *P* = .300) (Figure S4A and  B). Among patients who received non-IM treatments, there was no statistically significant sex difference in PFS or OS (Figure S4C and  D).

On multivariable analyses, males treated with IM had significantly lower OS (HR 1.47; 95% CI, 1.01–2.15; *P* = .044) and a trend towards lower PFS (HR 1.35; 95% CI, 0.95–1.94; *P* = .097) (Figure S5A and  B). Among patients who received non-IM treatment, there was no significant sex difference in PFS or OS (Figure S5C and  D). There was no significant interaction between sex and treatment group for PFS (*P* = .841) or OS (*P* = .525), consistent with no sex difference with IM treatment compared to non-IM treatment when adjusting for covariates.

## Discussion

Sex differences in GBM have garnered increasing attention over the last decade. Sex-biased anti-tumor immunity is considered an important underlying driver,^5^^,^^6^^,^^17^ though implications for treatment are unclear. In this single-institution retrospective study of patients with newly diagnosed or recurrent GBM enrolled on clinical trials, ICI treatment did not have a sex-specific effect on PFS or OS. Our *nGBM* analyses showed no statistically significant sex differences in PFS or OS within the ICI group or between the ICI and non-ICI groups, in both univariate and multivariable analyses. In *rGBM*, univariate analyses showed significantly worse PFS and a trend towards worse OS in males treated with ICI, while multivariable analyses showed significantly worse OS and a trend towards worse PFS in males treated with ICI. In all interaction analyses, associations between sex and survival outcomes did not differ significantly between ICI and non-ICI groups, indicating no sex-specific effect of ICI on PFS or OS in either *nGBM* or *rGBM*.

Increased cancer incidence and mortality in males have been observed across a range of malignancies.^18^ Despite this, male patients appear to derive greater therapeutic benefit from ICI in clinical trials spanning multiple systemic tumor types.^19^^,^^20^ Mechanisms that drive this sex difference are under investigation, and are believed to involve an interplay of anti-tumor immune response with genetic, hormonal, and metabolic factors acting on the tumor and the tumor microenvironment.^21^ In GBM, recent preclinical studies have demonstrated sex-based differences in anti-tumor immunity, including increased T-cell exhaustion in males and a survival benefit in male—but not female—mice treated with anti-PD1 therapy relative to control.^5^^,^^6^ In GBM patients, phase 3 clinical trials have failed to demonstrate efficacy of ICI treatment,^22-24^ but have not directly or prospectively evaluated sex-based differences. To our knowledge, this is the first clinical study to evaluate sex differences associated with immune checkpoint blockade or immunotherapy in GBM patients.

Contrary to the described preclinical findings, our results do not demonstrate improved survival for males with ICI treatment. In fact, *rGBM* males treated with ICI had worse OS and a trend towards worse PFS compared to females. Adjusted HRs among patients receiving non-ICI treatments also suggested numerically shorter PFS and OS in males, though these differences were not statistically significant. This pattern may reflect the underlying survival advantage for females previously observed in population studies of GBM.^2^ In our interaction analyses, ICI treatment did not meaningfully alter this baseline advantage. Given that putative mechanisms of sex-biased anti-tumor immunity in GBM are T-cell mediated, their relevance may extend to immunotherapies beyond ICI. However, our secondary analysis found no clear sex difference in PFS or OS associated with immunotherapy treatments collectively.

Our results are consistent with subgroup results from the Checkmate 498 and Checkmate 548 phase 3 trials of ICI in GBM. Though sex differences were not directly evaluated in these studies, reported HRs for death comparing treatment groups were either similar in males and females or trended towards worse survival in males. In Checkmate 498, HR for concurrent nivolumab (compared to temozolomide) was 1.24 (95% CI, 0.99–1.56) in males and 1.35 (95% CI, 0.99–1.84) in females.^23^ In Checkmate 548, HR for the combination of adjuvant temozolomide and nivolumab (compared to temozolomide and placebo) was 1.20 (95% CI, 0.93–1.54) in males and 0.96 (95% CI, 0.72–1.27) in females.^24^

Several factors may explain the discrepancy between our clinical findings and prior preclinical results. Mechanisms of tumor immunogenicity, immune regulation and hormonal interplay may differ meaningfully between genetically defined mouse models and genetically heterogeneous human patients. Surgical resection, corticosteroid use, and older age in human patients may also contribute to immunosuppression relative to mouse models. Additionally, mechanisms driving baseline sex differences in human GBM survival may exert stronger effects than the immunologic mechanisms identified in preclinical models. Finally, the lack of overall therapeutic efficacy of ICI in GBM may obscure sex-specific effects from becoming clinically apparent. Strengths of our study include evaluation of both nGBM and rGBM, evaluation of consecutive patients, large sample size, standardized data collection workflows for clinical trial patients, and adjustment for clinically relevant potential confounders in multivariable survival analyses. Our study also highlights the importance of using a reference group to evaluate sex-based treatment effects, as analyses of treated patients alone may be confounded by the baseline sex-based survival differences observed in GBM patients.^2^^,^^4^

Our study also has limitations. Retrospective design allows for detection of associations but not inference of causality, and the use of a single-institution clinical trial patient sample risks selection bias and limits generalizability. However, as ICI treatment is not FDA-approved for GBM, its use is largely limited to the clinical trial setting. Our analyses only allow us to draw conclusions about collective effects of ICI therapy rather than individual agents, though we note that anti-PD1 agents were most highly represented in our sample. The non-ICI treatment group included heterogeneous clinical trial treatments, and its strength as a reference group relies on the absence of sex-specific effects of these treatments. While no sex-based treatment effects are well established in GBM, potential sex differences associated with bevacizumab treatment in a subset of GBM have been proposed.^25^ Within constraints of our clinical study design, the non-ICI clinical trial population signifies a pragmatic reference group for patients treated with ICI on clinical trials. Finally, PFS and OS in GBM have unique limitations as endpoints,^26^ which prompted us to evaluate both. PFS may better assess the direct efficacy of a treatment, but may be confounded by pseudoprogression.^27-29^ While OS is often considered a gold-standard endpoint, it may be confounded by variability of interventions received after study treatment.

## Conclusions

In patients with newly diagnosed and recurrent GBM, ICI treatment was not associated with sex differences in PFS or OS. This finding persisted when immunotherapy treatments were analyzed collectively. Our findings are inconsistent with recent preclinical data which suggest that males may benefit preferentially from ICI treatment due to sex-based differences in anti-tumor immunity. Our study highlights the complex and multifactorial nature of sex differences in GBM patients, as well as the importance of evaluating preclinical findings in clinical studies. Future inquiry may focus on identifying subsets of patients for whom sex-biased immunologic mechanisms may lead to clinically meaningful differences in outcome. Given emerging biological mechanisms of sex differences in GBM, prospective evaluation of sex-based outcomes in future clinical trials may help translate these preclinical insights into improved therapies.

## Supplementary Material

vdaf250_Supplementary_Data

## Data Availability

The data that support the findings of this study are available from the corresponding author upon reasonable request.
