# Supplementary material for: Evaluation of sex differences in survival among glioblastoma patients treated with immune checkpoint inhibitors
Source: Neurooncol Adv. 2025 Dec 8;8(1):vdaf250. doi: 10.1093/noajnl/vdaf250 (PMC12901605; doi:10.1093/noajnl/vdaf250)
Supplement: vdaf250_Supplementary_Data [file vdaf250_supplementary_data.docx]

Supplementary Tables and Figures

**Table S1.** Treatments received on clinical trial by patients in the nGBM cohort.

|  | **Number of patients (%)** |
| --- | --- |
| **ICI** (n = 56) |  |
| Anti-PD1 | 45 (80%) |
| Anti-PDL1 | 9 (16%) |
| Anti-PD1 + anti-CTLA4 | 2 (4%) |
|  |  |
| **Non-ICI** (n = 240) |  |
| Kinase inhibitors | 90 (37%) |
| Temozolomide only | 36 (15%) |
| MIF pathway modulator | 33 (14%) |
| Peptide vaccine* | 34 (14%) |
| Anti-phosphatidylserine antibody* | 15 (6%) |
| PARP inhibitor | 11 (5%) |
| Other cytotoxic agents | 7 (3%) |
| Antibody-drug conjugate | 6 (3%) |
| Proteasome inhibitor | 3 (1%) |
| MDM2 inhibitor | 3 (1%) |
| Nuclear export inhibitor | 2 (1%) |

Non-ICI treatments classified as immunotherapy are denoted by an asterisk (*).

Abbreviations: *CTLA4* cytotoxic T-lymphocyte associated protein 4, *ICI* immune checkpoint inhibitor, *MDM2* mouse double minute 2 homolog, *MIF* macrophage migration inhibitory factor, *nGBM* newly diagnosed glioblastoma, *PARP* poly (ADP-ribose) polymerase, *PD1* programmed cell death protein 1, *PDL1* programmed death-ligand 1

**Table S2**. Treatments received on clinical trial by patients in the rGBM cohort.

|  | Number of patients (%) |
| --- | --- |
| **ICI** (n = 184) |  |
| Anti-PD1 | 150 (81.5%) |
| Anti-PDL1 | 15 (8%) |
| Anti-PD1 + anti-CTLA4 | 16 (9%) |
| Anti-LAG3 | 2 (1%) |
| Anti-LAG3 + anti-PD1 | 1 (0.5%) |
|  |  |
| **Non-ICI** (n = 274) |  |
| Kinase inhibitor  Viral, gene, or peptide-based immunotherapy* | 106 (39%)  40 (15%) |
| ISR inducer | 22 (8%) |
| MIF pathway modulator | 23 (8%) |
|  |  |
| Antiangiogenic agent | 22 (8%) |
| Cytotoxic agents | 19 (7%) |
| HIF pathway inhibitor | 11 (4%) |
| Nuclear export inhibitor | 9 (3%) |
| MDM2 inhibitor | 6 (2%) |
| Immunomodulators* (IDO1 inhibitor, CSF1R inhibitor, anti-CD137 agonist antibody) | 6 (2%) |
| Chemokine receptor antagonist | 3 (1%) |
| Antibody-drug conjugate | 2 (1%) |
| PARP inhibitor | 2 (1%) |
| Other targeted therapies (<1 % each) | 3 (1%) |

Non-ICI treatments classified as immunotherapy are denoted by asterisks (*).

Abbreviations: *CTLA4* cytotoxic T-lymphocyte associated protein 4, *HIF* hypoxia-inducible factor, *ICI* immune checkpoint inhibitor, *IDO1* indoleamine 2,3-deoxygenase, *ISR* integrated stress response, *LAG3* lymphocyte activation gene 3, *MDM2* mouse double minute 2 homolog, *MIF* macrophage migration inhibitory factor, *PARP* poly (ADP-ribose) polymerase, *PD1* programmed cell death protein 1, *PDL1* programmed death-ligand 1, *rGBM* recurrent glioblastoma


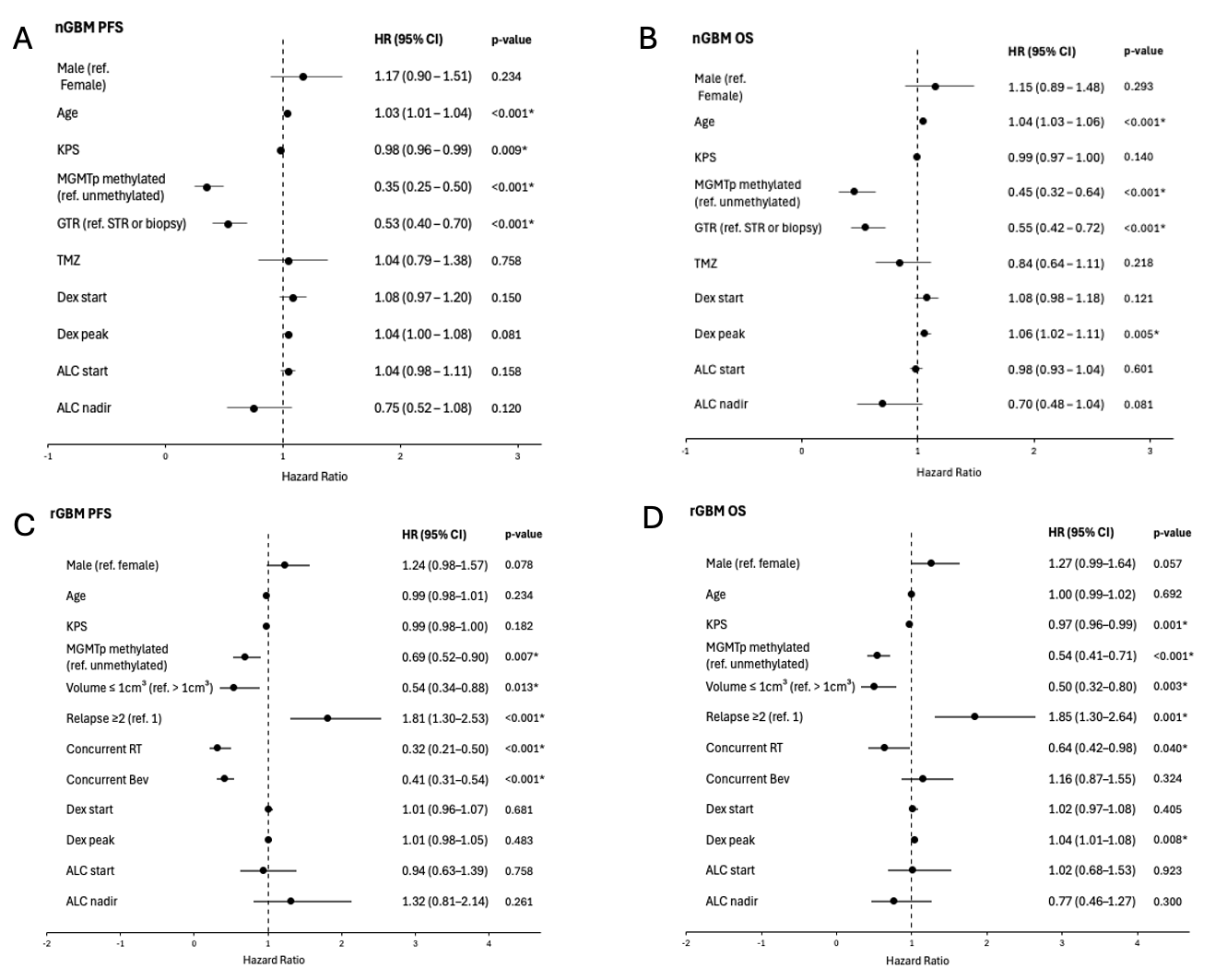


**Figure S1.** Forest plots of multivariable analyses for progression-free survival (PFS) and overall survival (OS). (A) PFS in newly diagnosed glioblastoma (nGBM), *IDH* wild-type. (B) OS in nGBM. (C) PFS in recurrent glioblastoma (rGBM), *IDH* wild-type. (D) OS in rGBM. P-values < 0.05 are denoted by asterisks (*).

Abbreviations: *ALC* absolute lymphocyte count, *bev* bevacizumab, *CI* confidence interval, *dex peak* dexamethasone peak dose (mg/day), *dex start* dexamethasone dose (mg/day) at start of treatment, *GTR* gross total resection, *IDH* isocitrate dehydrogenase, *KPS* Karnofsky performance score, *MGMTp* O6-methylguanine-DNA methyltransferase promoter, *ref.* reference, *RT* radiation therapy, *STR* sub-total resection, *TMZ* temozolomide concurrent and adjuvant treatment


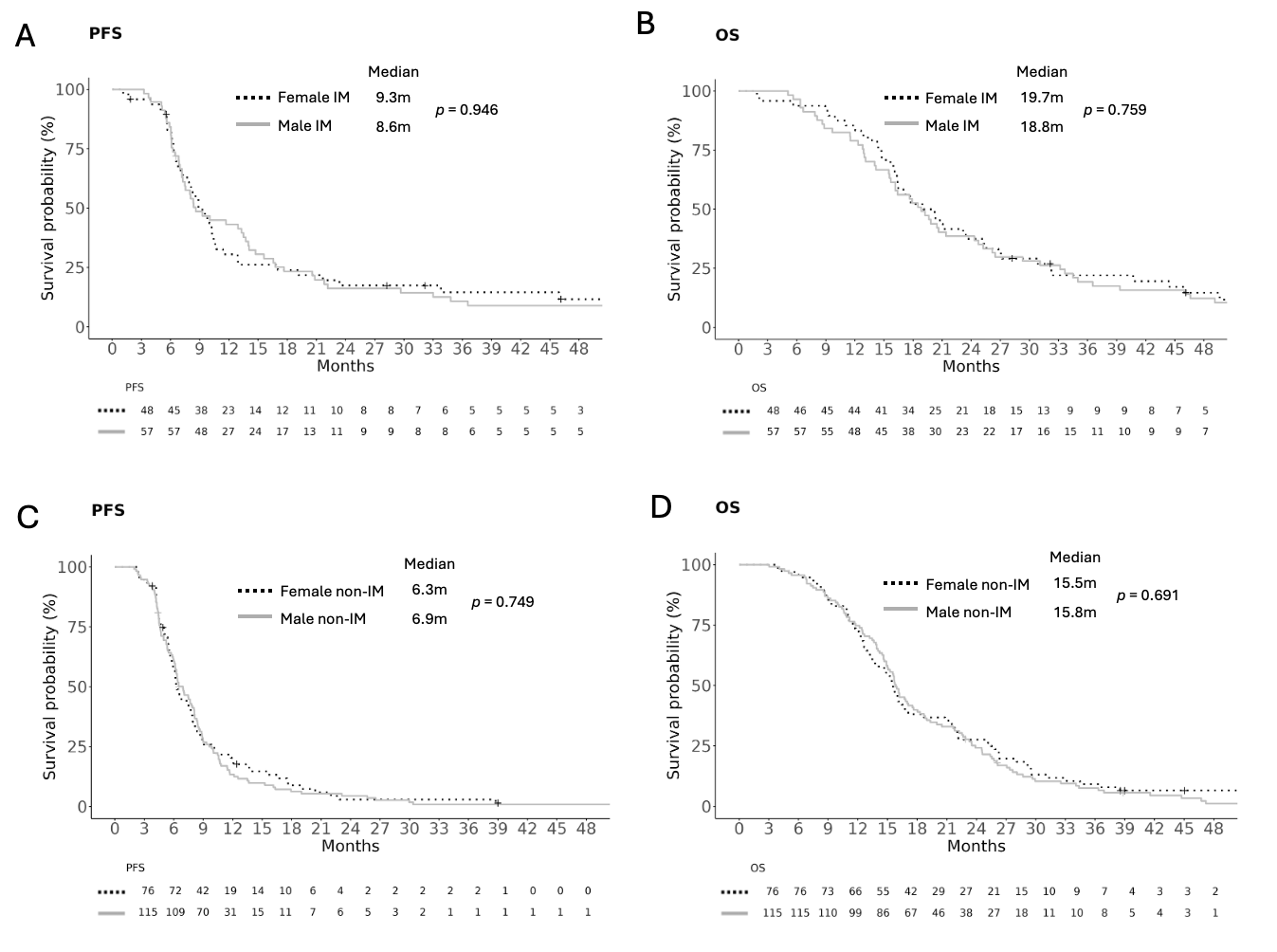


**Figure S2.** Kaplan-Meier analysis of progression-free survival (PFS) and overall survival (OS) in newly diagnosed glioblastoma (nGBM), *IDH* wild-type. (A) PFS in patients who received immunotherapy (IM). (B) OS in patients who received IM. (C) PFS in patients who received non-IM treatment. (D) OS in patients who received non-IM treatment. P-values from log rank tests are shown. Censored observations are denoted by “+” symbols.

Abbreviations: *IDH* isocitrate dehydrogenase, *m* months


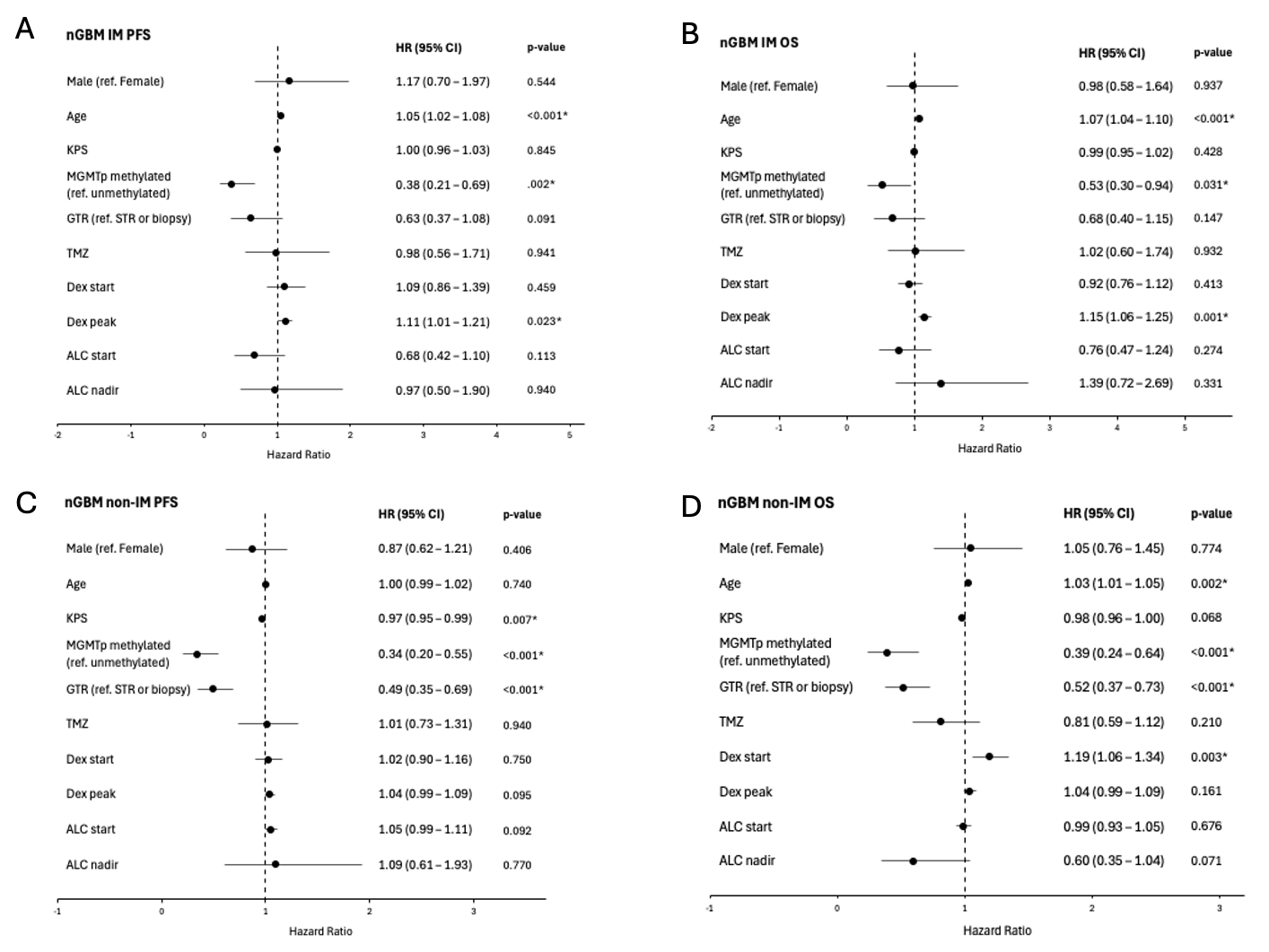


**Figure S3.** Forest plots of multivariable analyses for progression-free survival (PFS) and overall survival (OS) for newly diagnosed glioblastoma (nGBM), *IDH* wild-type. (A) PFS in patients who received immunotherapy (IM). (B) OS in patients who received IM. (C) PFS in patients who received non-IM treatment. (D) OS in patients who received non-IM treatment. P-values < 0.05 are denoted by asterisks (*).

Abbreviations: *ALC* absolute lymphocyte count, *CI* confidence interval, *dex peak* dexamethasone peak dose (mg/day), *dex start* dexamethasone dose (mg/day) at start of treatment, *GTR* gross total resection, *KPS* Karnofsky performance score, *MGMTp* O6-methylguanine-DNA methyltransferase promoter, *ref.* reference, *STR* sub-total resection, *TMZ* temozolomide concurrent and adjuvant treatment


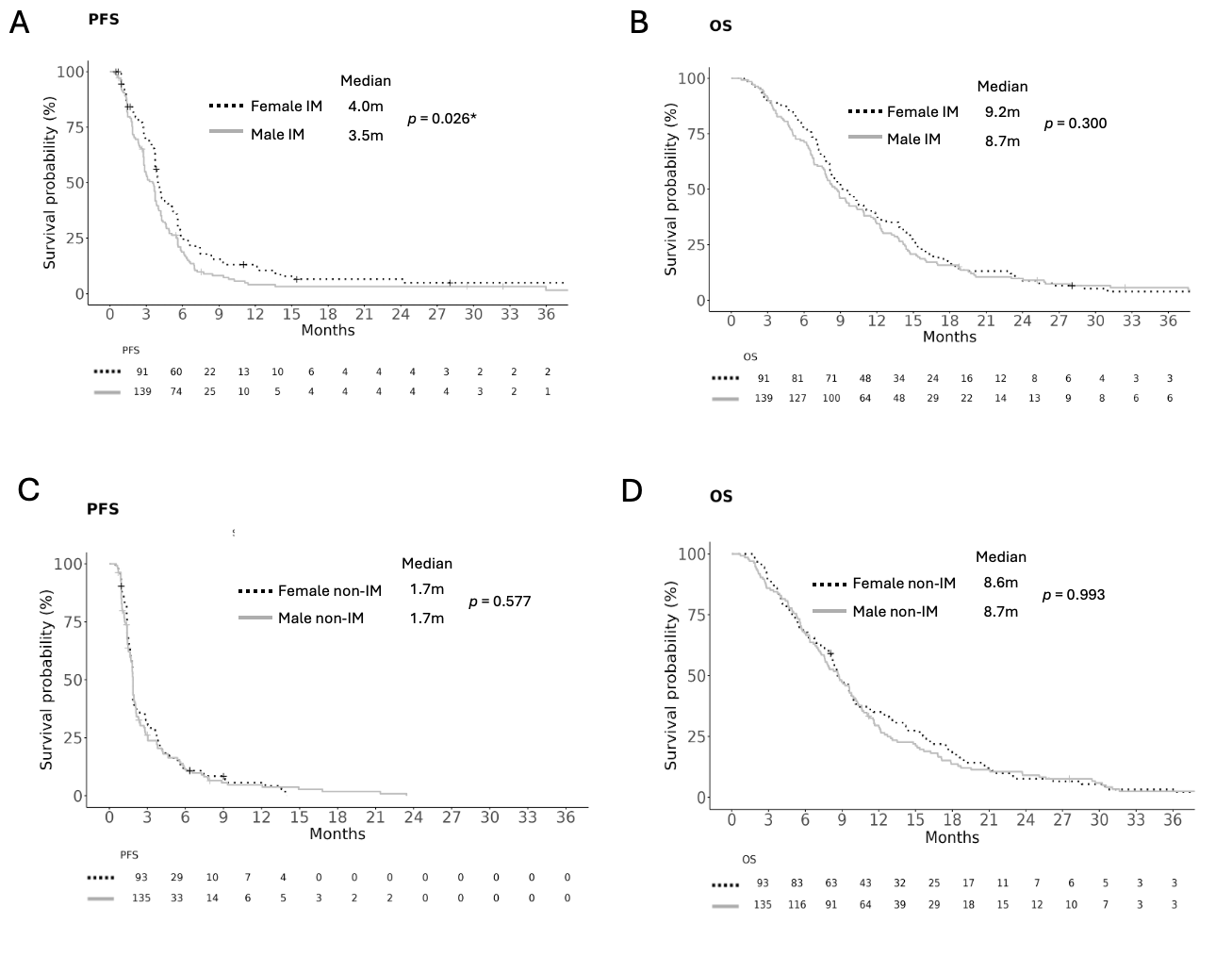


**Figure S4**. Kaplan-Meier analysis of progression-free survival (PFS) and overall survival (OS) in recurrent glioblastoma (rGBM), *IDH* wild-type. (A) PFS in patients who received immunotherapy (IM). (B) OS in patients who received IM. (C) PFS in patients who received non-IM treatments. (D) OS in patients who received non-IM treatments. P-values from log rank tests are shown. Censored observations are denoted by “+” symbols. P-values < 0.05 are denoted by asterisks (*).

Abbreviations: *IDH* isocitrate dehydrogenase, *m* months


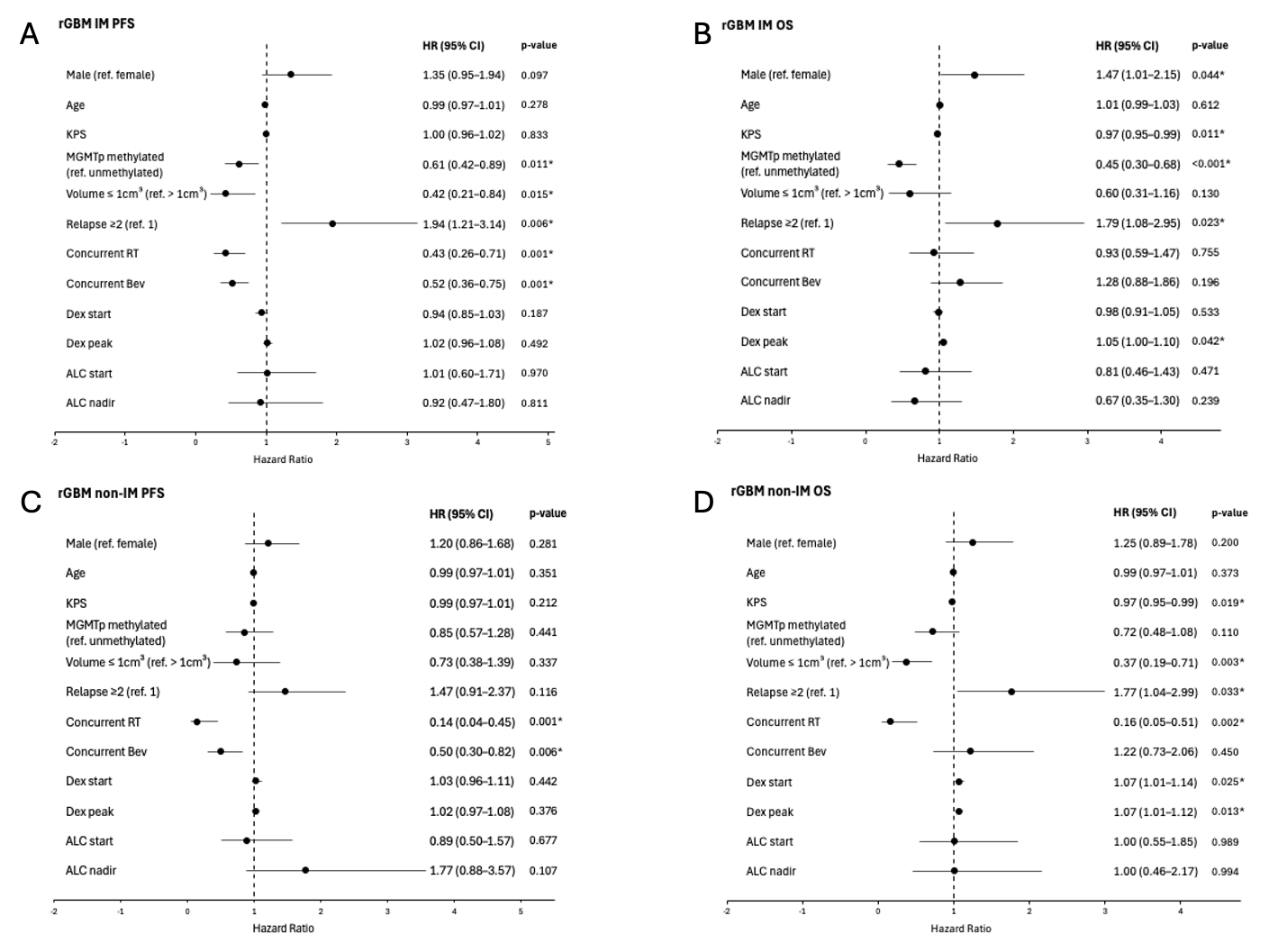


**Figure S5**. Forest plots of multivariable analyses for progression-free survival (PFS) and overall survival (OS) in recurrent glioblastoma (rGBM), *IDH* wild-type. (A) PFS in patients who received immunotherapy (IM). (B) OS in patients who received IM. (C) PFS in patients who received non-IM treatment. (D) OS in patients who received non-IM treatment. P-values < 0.05 are denoted by asterisks (*).

Abbreviations: *ALC* absolute lymphocyte count, *Bev* bevacizumab, *dex peak* dexamethasone peak dose (mg/day), *dex start* dexamethasone dose (mg/day) at start of treatment, *IDH* isocitrate dehydrogenase, *KPS* Karnofsky performance score, *MGMTp* O6-methylguanine-DNA methyltransferase promoter, *ref.* reference, *RT* radiation therapy
